# The association between derived TyG index and the risk of heart failure in the elderly population: a prospective cohort study from 2017 to 2023

**DOI:** 10.1186/s12889-025-22046-2

**Published:** 2025-03-04

**Authors:** Xinyang Dui, Xin Chen, Linlin Zhu, Xinyue Han, Tianpei Ma, Liang Lv, Guoyue Huang, Lin Hu, Jinyu Xiao, Zhuoma Diji, Nan Yang, Mengjie Hu, Jiaqiang Liao, Mengyu Fan, Xia Jiang, Tao Zhang, Jiayuan Li

**Affiliations:** 1 Department of Epidemiology and Biostatistics, West China School of Public Health and West China Fourth Hospital, Sichuan University, Chengdu, Sichuan China; 2 Department of Toxic Nephrology, West China Fourth Hospital, Sichuan University, Chengdu, Sichuan China; 3 The Johns Hopkins University, Bloomberg School of Public Health, Baltimore, USA; 4 West China Institute of Preventive and Medical Integration for Major Diseases, Sichuan University, Chengdu, Sichuan China; 5 Department of Nutrition and Food Hygiene, West China School of Public Health and West China Fourth Hospital, Sichuan University, Chengdu, Sichuan China; 6 West China-PUMC C.C. Chen Institute of Health, Sichuan University, Chengdu, Sichuan China

**Keywords:** Heart failure, Derived TyG index, Prospective cohort study

## Abstract

**Background:**

Diabetes and obesity are established risk factors for heart failure(HF). Although the TyG (triglyceride-glucose) index serves as a sensitive marker for identifying insulin resistance, there is a lack of comprehensive evidence regarding whether its integration with obesity indices can enhance the predictive capacity for HF. This prospective cohort study is designed to explore the correlation between TyG indices in conjunction with obesity indices (TyG-body mass index, or TyG-BMI; TyG-waist circumference, or TyG-WC; TyG-waist circumference-to-height ratio, or TyG-WHtR) and the risk of HF.

**Methods:**

Between 2017 and 2023, the study employed a prospective cohort study design to investigate all older adults aged 60 years and above who completed at least twice periodical health examinations in the National Basic Public Health Service at the Hongguang Community Health Service Center. The association between TyG and its derived indices (TyG-BMI; TyG-WC; TyG-WHtR) and the risk of HF was assessed by Cox modelling, as well as their longitudinal trajectories fitted using a group-based trajectory model.

**Results:**

A total of 7,335 people participated in the study. During an average follow-up period of 2.97 years, 229 participants were eventually diagnosed with HF. Findings showed that individuals with a TyG-BMI less than 142 or greater than or equal to 169, TyG-WC greater than or equal to 614, and TyG-WHtR greater than or equal to 3.85 had a higher risk of developing HF, with hazard ratios (*HR*) and 95% confidence intervals (*CI*s) of 1.17 (1.15, 2.55), 1.45 (1.06. 1.98), 1.54 (1.09, 2.18) and 1.33 (1.01, 1.75). In terms of trajectories, the three derived indexes exhibited relatively stable fluctuations. Specifically, among men, those with low-level fluctuations in the TyG-BMI trajectory had a hazard ratio of 2.37 for HF compared to those with a medium-level wave.Compared to individuals whose TyG-WHtR levels fluctuate around 3.71 over five years, those with TyG-WHtR levels approaching 3.29 and steadily decreasing face an 80% higher risk of developing HF. However, there was no such difference observed in women.

**Conclusion:**

This study demonstrates a difference in the risk of HF among populations with varying levels of TyG combined with obesity indicators. In addition, persistently low and decreasing levels of TyG-WHtR also indicate an increased risk of developing HF. These biomarkers can be used as effective practical tools for identifying those at high risk of developing HF in the community’s older population.

**Graphical Abstract:**

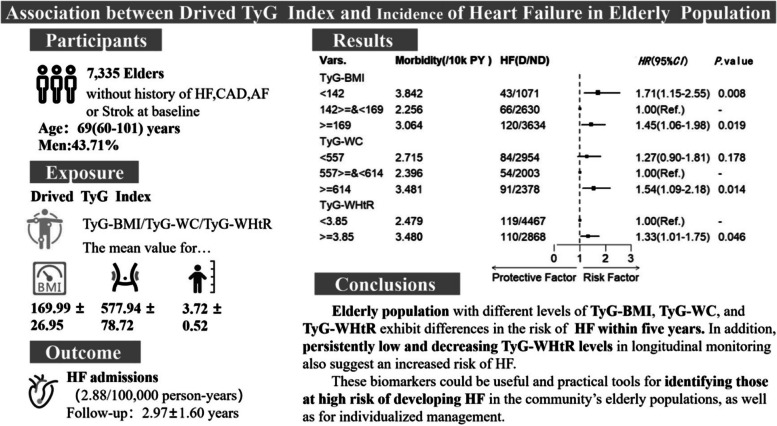

**Supplementary Information:**

The online version contains supplementary material available at 10.1186/s12889-025-22046-2.

## Key points

**Question:** Whether TyG combined with obesity index is associated with the risk of heart failure in elderly people in the community?

**Findings:** This prospective cohort study based on a community-based elderly population found a strong association between TyG-BMI, TyG-WC, and TyG-WHtR and the risk of developing HF.

**Meaning:** The derived TyG index can be used as an effective biomarker to identify the risk of heart failure in older adults within 5 years.

**Supplementary Information:**

The online version contains supplementary material available at 10.1186/s12889-025-22046-2.

## Background

Heart failure (HF) is a cardiovascular disorder characterized by structural and/or functional abnormalities of the heart. It represents an advanced stage of various cardiovascular diseases and is associated with high rates of disability and mortality [1]. About 40 million people worldwide suffer from heart failure, and the disease has a death rate of more than 10% [2]. It is estimated that 12.1 million people in China alone have been diagnosed with HF, and the number of cases is rising [3, 4]. Heart failure is more common in the elderly; incidence rates for those 65–79 years old and those over 80 years old are 892 and 1,655 per 100,000 person-years, respectively [3], and the prevalence of “various forms of heart failure” is approximately 11.8% [5]. Since age-related factors make people more susceptible to disease, cardiovascular aging-related physiologic changes make people more susceptible to heart failure [6]. Older individuals may lack the typical symptoms of heart failure, which causes more complications, easy misdiagnosis, and missed diagnosis [3].

The American Heart Association/American College of Cardiology/Heart Failure Society of America (AHA/ACC/HFSA) have focused on heart failure prevention in their latest heart failure guidelines released in 2022 and have recommended the use of heart failure risk prediction models to identify those at risk. Notably, diabetes and obesity have been included as primary indications for HF prevention in the new guidelines.

The development of HF is closely linked to insulin resistance and obesity through several different physiological pathways. In response to injury, the myocardium typically modifies substrate metabolism to boost energy efficiency. However, insulin resistance disrupts this adaptive process, potentially exacerbating damage through factors such as lipotoxicity, inflammation, oxidative stress, and fibrosis [7]. And obesity can lead to heart failure by altering cardiac dynamics, structure, function, and conduction [8]. Compared to the homeostatic model assessment of insulin resistance (HOMA-IR), the TyG index is more sensitive [9] and easier to obtain, making it an effective alternative indicator for identifying insulin resistance in large-scale epidemiological studies [10]. Furthermore, the TyG index has been confirmed to be positively associated with the risk of HF occurrence and may serve as a crucial therapeutic target and significant predictive indicator for HF [11, 12]. However, previous research evidence has been predominantly based on cross-sectional studies. The TyG index combined with obesity indicators (triglyceride-glucose-body mass index, or TyG-BMI; triglyceride-glucose-waist circumference, or TyG-WC; triglyceride-glucose-waist-to-height ratio, or TyG-WHtR), collectively known as TyG-derived indices, can comprehensively reflect the risk of diabetes and obesity in the population and have demonstrated superior performance in predicting and diagnosing various cardiovascular diseases, such as coronary artery calcification progression [13] and ischemic stroke [14], compared to the TyG index alone.

Currently, there is limited research on the association between derived TyG indices and HF, with the majority of studies focusing on specific populations and ignoring the geriatric population [15–17]. It is worth noting that for most older people, lack of awareness of physical and hypertensive symptoms and delayed emotional responses prevent them from seeking timely medical help [17]. Therefore, it is imperative to conduct research and develop straightforward, reliable markers to accurately and sensitively warn of the elderly’s risk of HF.

The aim of this study was to investigate the association between the TyG index and the risk of HF in the elderly population over a five-year period, using periodical health examinations data from the regional elderly population, in order to provide insightful information for the development of clinical procedures and public health policies for the elderly.

## Research design and method

### Research design

This study utilizes data from the Hongguang Elderly Health Examination Cohort. China’s National Basic Public Health Service Project began providing annual health examinations to those 65 years of age and above in 2009. Since 2017, Chengdu Pidu District Hongguang Community Health Service Center has standardized the management of physical examination data collected by the National Basic Public Health Service Project, and an independent database has been formed.

Participant recruitment was conducted in Hongguang Town, Pidu District, Chengdu City, Sichuan Province. Hongguang Town, a suburban area of Chengdu, is undergoing urbanization, and therefore, this region encompasses both urban and rural representative populations. The cohort comprises 12,577 adults aged around 60 and above from 14 communities, representing 47.43% of the local age-eligible population. Baseline data were collected through health check-ups, including demographic information, general health examinations, lifestyle factors, health status and medication use, and health assessments. Disease outcomes were confirmed by linking health examination data with the disease diagnosis information system on the first page of medical records managed by the Sichuan Provincial Health Information Center. A detailed description of the cohort has been showed in Additional file 1.

**Fig. 1:**
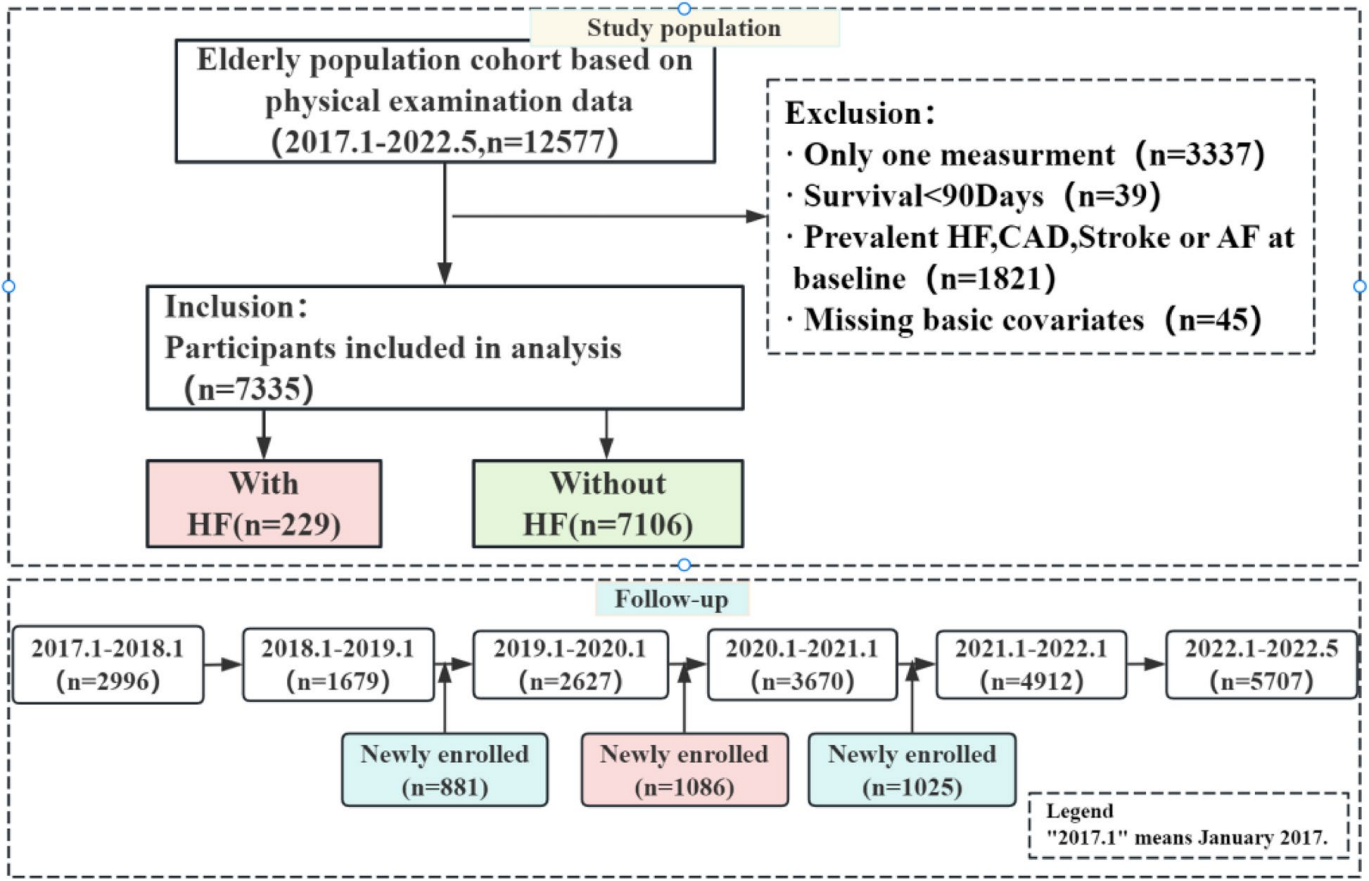
Flowchart of study participants and follow-up

The study included elderly people who participated in China’s National Basic Public Health Service Project between January 2017 and January 2023. To ensure regular participant engagement in follow-up assessments and to guarantee the reliability of outcome data collection, this study excluded participants with only one health measurement. The study procedure is illustrated in Fig. 1. Exclusion criteria comprised the following:(1) participants with only once measurement;(2) participants with a survival time of less than 90 days;(3) participants with missing data about covariates of interest;(4) participants who developed heart failure or those who have previous coronary heart disease, stroke, or atrial fibrillation at baseline.

### Data collection

Prospective cohort data collection involved gathering information on health-related behavioral habits, primarily focusing on smoking status and alcohol consumption. Health history surveys were conducted, including family history, surgical history, and medication allergies. The health examination indicators primarily included height, weight, blood pressure, fasting blood glucose (FPG), total cholesterol (TC), triglycerides (TG), and high-density lipoprotein cholesterol (HDL-C). Specific measurement methods for the main research variables are provided in Additional File 2.

This study was approved by Ethics Committee of West China Fourth Hospital and West China School of Public Health, Sichuan University, and all participants provided written informed consent.

### Diagnostic criteria

This study matched the health examination data of the research subjects with the disease diagnosis information system on the first page of medical record through the Sichuan Provincial Health Information Center, collected the first diagnosis information of the participants’ research outcomes, and confirmed the occurrence and outcome of the participants’ diseases. The diagnosis of incident heart failure (HF) was defined as the presence of an HF diagnosis (ICD-10,International Classification of Diseases-10, code: I50) in the participants’ hospital admission records during the follow-up period.

The endpoint of patient follow-up was either the date of the HF diagnosis or the date of the last medical examination, whichever came earlier. The diagnosis of hypertension was defined as a clear diagnosis of hypertension (ICD-10 codes: I10-15) in the participants’ hospital admission records during the follow-up period. If a participant developed HF during the follow-up period, the date of hypertension diagnosis should be earlier than the date of HF diagnosis.

The TyG, TyG-BMI, TyG-WC, and TyG-WHtR indices were calculated using the following formulas: (1) TyG = ln[triglycerides(mg/dL) × fasting blood glucose(mg/dL)/2]; (2) BMI = weight (kg) / height^2^ (m^2^); (3) WHtR = waist circumference (cm) / height (cm); (4) TyG-WC = TyG × waist circumference (cm); TyG-WHtR = TyG × WHtR; TyG-BMI = TyG × BMI.

### Statistical methods

Before commencing the statistical analysis, we conducted a data quality check and imputed some missing values using the random forest method. The participants were grouped according to the occurrence of HF by the end of follow-up, and baseline information was compared between the two groups. Continuous variables were reported as mean ± standard deviation(*x* ± *s*), and independent sample *t*-tests were used for between-group comparisons. Categorical variables were presented as proportions, and *Chi-square* tests were performed for between-group comparisons. The non-linear relationship between the derived TyG indices and HF was evaluated using the restricted cubic spline (RCS) model. The optimal number of knots was determined based on the Akaike information criterion (*AIC*). Cumulative survival time of HF at different levels of the derived TyG indices was calculated and compared using the Kaplan–Meier method and log-rank test. The Cox proportional hazards regression models were utilized to investigate the correlation between the exposure factors (categorized according to the RCS cutoff value) and HF. Additionally, the cross-product interaction term in the Cox model and relative excess risk estimation (RERI) were used to examine the potential multiplicative or biological interaction between obesity and the TyG index in relation to HF risk. Further details of the interaction results are provided in Additional file 2:Table S1.

Joint and subgroup analyses were conducted by age, history of hypertension, smoking, alcohol consumption, and gender, considering that individuals with different characteristics have distinct risks of HF [18]. Previous epidemiological surveys have shown that the incidence of heart failure is higher in the elderly, patients with hypertension, and female populations.

Using a group-based trajectory model (GBTM, a finite mixture model), gender-specific longitudinal data of the derived TyG index were fitted to two or more latent trajectories using maximum likelihood.We grouped the study population according to GBTM fitting results to clarify the impact of fluctuations and trajectories of these indices on the risk of heart failure in older adults and to complement the results of prospective exploratory studies based on single-measurement data. The Cox regression model was used to analyze the relationship between different longitudinal trajectories and the probability of new-onset HF, using the moderate-level group as the reference group. (Refer to the Third Part of Additional file 2 for more details on the trajectory model).

Data analysis was performed using R language software 4.2.3, and SAS 9.4 was used to fit the GBTM trajectory of the Proc Traj program developed by Nagin et al. A two-sided test was used, and *P* < 0.05 was considered a statistically significant difference.

## Results

### General characteristics of the study population

The characteristics of the participants in the two groups, with the occurrence of heart failure as the endpoint, are shown in Table 1. After excluding participants who failed to meet the criteria, a total of 7,335 participants were included in the analysis.The average age of the study participants was 69 years, with males accounting for 43.71%(3206/7335) of the cohort. By the end of the follow-up period, 229 participants were newly diagnosed with heart failure. The mean values for TyG, TyG-BMI, TyG-WC, and TyG-WHtR among the study subjects were 7.01, 169.99, 577.94, and 3.72, respectively. Individuals who eventually developed HF were more likely to be older, with higher levels of waist circumference, waist-to-height ratio, TyG-WC, TyG-WHtR, and serum creatinine (Scr), and were at a higher risk of developing hypertension. However, they had lower levels of height and hemoglobin(Hb) compared to individuals who did not develop HF.

**Table 1:** Description of baseline characteristics of the study population

Variable Names	Total Subjects	Non-HF Group	HF Group	*P*-value
HF[%(n/N)]	100 (7335/7335)	96.88(7106/7335)	3.12(229/7335)	—
Male[%(n/N)]	43.71(3206/7335)	43.78(3111/7106)	41.48(95/229)	0.534
Age(*x* ± *s*,years)	68.99 ± 5.33	68.9 ± 5.28	71.78 ± 6.02	< 0.001
Height(*x* ± *s*,cm)	155.47 ± 8.15	155.51 ± 8.15	154.14 ± 8.01	0.011
BMI(*x* ± *s*,kg/m^2^)	24.21 ± 3.11	24.20 ± 3.09	24.45 ± 3.55	0.300
WHtR(*x* ± *s*)	0.53 ± 0.06	0.53 ± 0.06	0.55 ± 0.06	< 0.001
WC(*x* ± *s*,cm)	82.36 ± 8.62	82.30 ± 8.61	84.08 ± 8.98	0.003
SBP(*x* ± *s*,mm Hg)	139.26 ± 17.58	139.18 ± 17.57	141.68 ± 17.97	0.030
DBP(*x* ± *s*,mm Hg)	83.17 ± 11.29	83.20 ± 11.31	82.48 ± 10.68	0.188
IR-related Indicators(*x* ± *s*)				
TyG	7.01 ± 0.47	7.01 ± 0.47	7.00 ± 0.46	0.887
TyG_BMI	169.99 ± 26.95	170.01 ± 26.92	172.36 ± 29.84	0.397
TyG_WHtR	3.72 ± 0.52	3.72 ± 0.52	3.83 ± 0.54	0.002
TyG_WC	577.94 ± 78.72	577.56 ± 78.58	589.73 ± 82.28	0.028
Co-existing Diseases[%(n/N)]				
T2D	2.88(211/7335)	2.86(203/7106)	3.49(8/229)	0.714
HP	6.63(486/7335)	6.37(453/7106)	14.41(33/229)	< 0.001
Unhealthy Lifestyle[%(n/N)]				
Smoking History	26.39(1936/7335)	26.50(1883/7106)	23.14(53/229)	0.290
Drinking History	30.92(2268/7335)	31.10(2210/7106)	25.33(58/229)	0.074
Laboratory Indicators				
TC(*x* ± *s*,mmol/L)	5.12 ± 0.94	5.13 ± 0.95	5.06 ± 1.00	0.286
TG(*x* ± *s*,mmol/L)	1.43 ± 0.60	1.43 ± 0.61	1.43 ± 0.60	0.850
Scr(*x* ± *s*,μmoI/L)	63.58 ± 14.50	63.49 ± 14.42	66.21 ± 16.59	0.015
LDL-C(*x* ± *s*,mmol/L)	2.76 ± 0.73	2.75 ± 0.73	2.72 ± 0.75	0.821
HDL-C(*x* ± *s*,mmol/L)	1.49 ± 0.33	1.49 ± 0.33	1.45 ± 0.33	0.057
FPG(*x* ± *s*,mmol/L)	94.70 ± 15.12	95.02 ± 15.50	94.13 ± 16.02	0.289
TBIL(*x* ± *s*,μmoI/L)	11.45 ± 5.10	11.44 ± 5.07	11.59 ± 5.58	0.408
Hb(*x* ± *s*,g/L)	140.27 ± 24.30	140.36 ± 24.46	137.58 ± 18.91	0.046
WBC(*x* ± *s*, × 10^9^/L)	6.19 ± 1.97	6.18 ± 1.97	6.29 ± 1.82	0.188
PLT(*x* ± *s*, × 10^9^/L)	174.86 ± 67.14	175.02 ± 66.96	170.40 ± 72.21	0.629
ALT(*x* ± *s*,U/L)	22.64 ± 15.75	22.58 ± 15.38	24.91 ± 24.44	0.255
AST(*x* ± *s*,U/L)	27.00 ± 16.27	26.95 ± 16.15	28.60 ± 18.84	0.272

*Abbreviations*: *HF* Heart Failure, *BMl* Body Mass Index, *WHtR* Waist-to-Height Ratio, *WC* WaistCircumference, *SBP* Systolic Blood Pressure, *DBP* Diastolic Blood Pressure, *T2D* Type 2 Diabetes, *HP* Hypertension, *TC* Total Cholesterol, *TG* Triglycerides, *Scr* Serum Creatinine, *LDL-C* Low-Density Lipoprotein Cholesterol, *HDL-C* High-Density Lipoprotein Cholesterol, *FPG* Fasting Plasma Glucose, *TBIL* Total Bilirnbin, *Hb* Hemoglobin, *WBC* White Blood Cell Cout, *PLT* Platelet Count, *ALT* Alanine Aminotransferase, *AST* Aspartate Aminotransferase

### Nonlinear relationship between derivative TyG indices and the risk of HF

TyG-BMI and TyG-WC indices showed a significant non-linear dose–response association with HF (*P*_overall trend_ < 0.05, *P*_non-linearity_ < 0.05), while no such association was observed between TyG, TyG-WHtR and HF (*P*_overall trend_ > 0.05). Detailed results are shown in the Additional file 2: Figure S2.

### Association between TyG indices and HF incidence in elderly populations

#### Survival analysis

Significant differences were observed in the cumulative survival time across different groups of TyG-BMI, TyG-WC, and TyG-WHtR (*P* < 0.05), referring to Additional file 2: Figure S3 the Kaplan–Meier curve. Cox regression analysis showed that, after adjusting for age, gender, smoking, alcohol consumption, history of hypertension, scr, SBP, DBP, HDL-C, and LDL-C, the impact of different levels of TyG on the risk of HF did not reach statistical significance. However, individuals with TyG-BMI levels below 142 or greater than or equal to 169, as well as those with TyG-WC levels greater than or equal to 614, had a significantly increased risk of developing HF (hazard ratio = 1.71, 1.45, and 1.54, respectively). Moreover, individuals with TyG-WHtR $\ge$ 3.85 had a 33% significantly increased risk of HF (Table 2).

**Table 2:** Survival analysis of TyG and derived TyG indices in relation to HF

Model Parameters	Morbidity (/10,000 Person Years)	Bivariate Mode	Adjust Model
		*HR*(95%*CI*)	*HR*(95%*CI*)
TyG-Q			
< 7	2.958	Reference	Reference
> = 7	2.791	0.87(0.66–1.16)	0.92(0.70–1.22)
TyG-BMI-Q			
< 142	3.842	1.71(1.16–2.51)	1.71(1.15–2.55)
142 > = & < 169	2.256	Reference	Reference
> = 169	3.064	1.45(1.07–1.96)	1.45(1.06–1.98)
TyG-WC-Q			
< 557	2.715	1.22(0.87–1.72)	1.27(0.90–1.81)
557 > = & < 614	2.396	Reference	Reference
> = 614	3.481	1.63(1.15–2.29)	1.54(1.09–2.18)
TyG-WHtR-Q			
< 3.85	2.479	Reference	Reference
> = 3.85	3.480	1.47(1.13–1.91)	1.33(1.01–1.75)

Adiust Model: Adjusted for age, gender, history of hypertension, smoking history, alcohol consumption, Serum creatinine, Systolic Blood Pressure, Diastolic Blood Pressure, High-Density Lipoprotein Cholesterol and Low-Density Lipoprotein Cholesterol

*Abbreviations: D/ND* Disease/Non-disease

#### Joint analysis of the derived TyG index and traditional risk factors

**Fig. 2:**
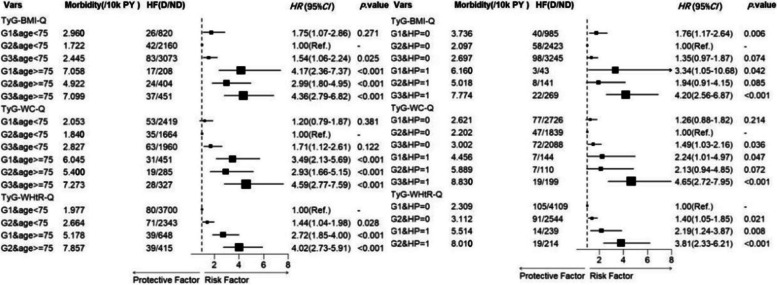
Forest plot of joint analysis based on age and history of hypertension. Abbreviations:G1:Group1(TyG-BMI: < 142;TyG-WC: < 557;TyG-WHtR: < 3.85);G2:Group2(TyG-BMI:142 >  = & < 169;TyG-WC:557 >  = & < 614;TyG-WHtR: >  = 3.85); G3: Group 3(TyG-BMI: >  = 169;TyG-WC: >  = 614)

The joint analysis revealed that the combined effects of age and hypertension significantly elevated the risk of HF in relation to derived TyG indices. Specifically, the highest risk of HF (*HR* and 95% *CI*: 4.36, 2.76–6.82) was observed in older individuals with a high TyG-BMI values compared to the reference group, while the impact of other indicators remained relatively consistent. Older adults aged 75 and above with higher TyG-WC and TyG-WHtR had *HR* and 95% *CI* values of 4.59 (2.77–7.59) and 4.02 (2.73–5.91), respectively, compared to the reference group. Moreover, hypertension history exhibited a similar effect as age on the association between derived TyG index and the risk of HF. The specific results of the joint analysis on the derived TyG index, age, and history of hypertension can be found in Fig. 2.

Individuals with a history of smoking and abnormal levels of derived TyG indices (e.g., TyG-BMI, TyG-WC, or TyG-WHtR) were at a significantly higher risk of developing HF compared to those with smoking history or abnormal TyG indices alone. Compared to individuals with no history of smoking and moderate levels of TyG-BMI, those with lower levels of TyG-BMI and a smoking habit had a higher risk of developing HF (*HR* and 95% *CI*: 2.35, 1.30–4.26). Additionally, individuals with higher levels of TyG-WC and TyG-WHtR who smoked had the highest risk of HF compared to the reference group (with *HR* and 95% *CI* values of 2.00, 1.22–3.26 and 2.00, 1.26–3.17, respectively). The specific results of the joint analysis on the derived TyG index and history of somking/drinking are provided in Additional file 2: Figure S4.

#### Subgroup analysis by gender

**Fig. 3:**
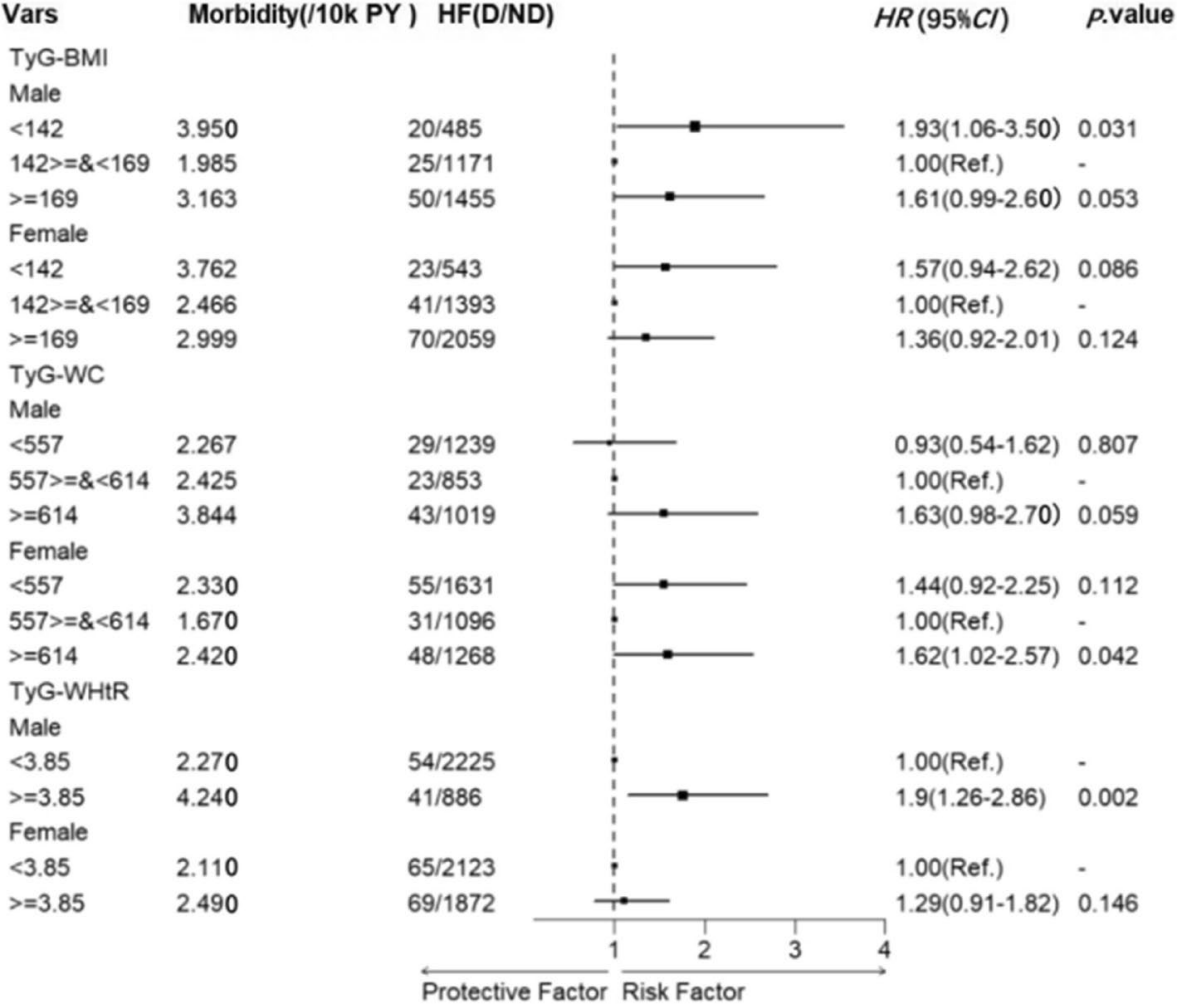
Forest plot of subgroup analysis by gender for TyG-derived indices

The trends between TyG-derived indices and the risk of HF were found to be consistent with the overall analysis, according to sex-specific subgroup analysis. In particular, the TyG-BMI and TyG-WHtR indices were more sensitive in the male population, while the female population showed higher sensitivity for TyG-WC indices. See Fig. 3 for detailed results.

## Discussion

This research examined the association between TyG-BMI, TyG-WC, and TyG-WHtR with the risk of heart failure (HF) using longitudinal health examination data from senior citizens. Significant correlations between these variables were found, with different correlations for different genders. Additionally, there was a combined influence of age, a history of hypertension, and unhealthy lifestyle with calculated TyG indices. Gender variations were also observed in the connection between TyG-derived indices and HF. The level of the study population’s derived TyG indexes remained rather stable throughout the five-year follow-up, as they are dependable indicators for determining the older population’s risk of HF. However, the trajectory of TyG-WHtR was slightly different in men, and those with a low TyG-WHtR index and continuous decrease had a higher risk of HF. These findings underscore the importance of simultaneously monitoring individuals with low levels and declining trends in TyG-WHtR over time, as they may be particularly susceptible to an increased risk of HF. No similar phenomenon was observed in women.

### Associations between derived TyG indexes and HF

Consistent with previous research findings, the combined indices of TyG with BMI, WC, and WHtR in this investigation demonstrated a stronger connection with the risk of incident HF in older population than did TyG alone. The TyG-WC index outperformed the TyG index in predicting the risk of stroke (with AUC,Area Under Curve, values of 0.622 and 0.614, respectively), according to a study based on a rural cohort in China [19]. In addition, a different longitudinal investigation found that, in comparison to the TyG and TyG-BMI indices, the TyG-WC index was more predictive of the development of coronary artery atherosclerosis [13].

According to a cohort study on the Korean population [20], TyG-WC and TyG-WHtR were more predictive of cardiovascular illnesses in non-diabetic adults than TyG and TyG-BMI. While WC and WHtR are indications of abdominal obesity, BMI is an indicator of overall obesity. Combining TyG with obesity indices, the TyG-BMI, TyG-WC, and TyG-WHtR indices offer a thorough representation of the effects of obesity and insulin resistance on population health. These indicators are linked to potential risk factors for heart failure. Endothelial dysfunction brought on by insulin resistance inhibits nitric oxide synthesis, aggravating hypoxia and ultimately leading to cardiac cell death [21]. Additionally, obesity and insulin resistance lead to oxidative stress, chronic inflammation, and abnormal lipid metabolism. These factors cause myocardial remodeling and fibrosis, which worsen heart failure [22, 23].

Previoues Research on the nonlinear association between cardiovascular events and the TyG-BMI index has produced contradictory results [14, 24–26]. Some studies found no nonlinear relationship between TyG-BMI and the severity of ischemic stroke [14] or coronary artery disease [26]. However, a nonlinear association has been found between TyG-BMI and the risk of stroke according to a study that focused on Chinese individuals [25]. Specifically, the risk of stroke in people with TyG-BMI < 174.63 is more likely to be affected by TyG-BMI. Our study provides support for the existence of a nonlinear relationship between TyG-derived indices and cardiovascular events. Compared with the previous study [25], the average age of our study population was older, the overall TyG-BMI level was lower, and the cut-off value was lower than 174.63. From these two studies of Chinese populations, it appears that, the derived TyG index had a greater impact on the risk of cardiovascular events among those with lower TyG-BMI levels.

A non-linear relationship between BMI and the death rate from cardiovascular disease was discovered by a meta-analysis based on prospective studies [27], while studies looking at the relationship between waist circumference and major cardiovascular events [28] also revealed similar results. Our study also suggested a nonlinear relationship between TyG combined with BMI and WC and the risk of HF. Considering the unique nature of BMI and WC indicators, more investigation is required to validate the dose–response association between TyG-BMI, TyG-WC, and the risk of cardiovascular disease. Furthermore, there are two phases of slow increase and rapid increase in the rate of heart failure incidence with increasing TyG-WHtR, indicating that the link between the TyG-WHtR index and the risk of heart failure is not simply linear. Thus, specific intervention strategies adapted to various phases of derived TyG indexes could improve heart failure prevention.

Notablely, contrary to several other studies on the general population, our investigation did not find a relationship between the TyG index and the risk of heart failure in the older population [11]. This implies that the TyG index might not be a reliable gauge of heart failure risk in the older population. Older population may experience more health issues and physiological changes, such as vascular aging and a decline in myocardial function. These variables may combine to affect the TyG index in a way that makes it less predictive for older adults and makes it unable to adequately explain the risk of heart failure. Future studies must thus concentrate more on the physiological traits of the older population as well as the variations in the etiology of heart failure.

Our study also delved into the potential interaction between these obesity indicators and the TyG in relation to HF risk and suggested that there is no significant synergy or interaction between these factors in their impact on HF risk. A study focused on the interaction of the TyG index and obesity for stroke risk results is consistent with this study [29]. While both types of indicators are associated with metabolic disorders, the pathogenesis of heart failure may be different. Further studies are needed to elucidate the specific mechanisms through which obesity and insulin resistance contribute to heart failure.

### Role of age, hypertension, unhealthy lifestyle and sex

The results of the joint analysis demonstrated that derived TyG indices had a negative impact on the risk of heart failure and were more deleterious in older adults, those with a history of hypertension, smokers, and alcohol consumers. This shows that preventing smoking and alcohol intake, keeping blood pressure within normal ranges, and doing regular check-ups on senior people can all help to lessen the cumulative impact of these factors and potentially prevent heart failure. The results of the subgroup analysis showed that different gender groups had varying levels of sensitivity to TyG-derived indices. In particular, TyG-BMI and TyG-WHtR had superior predictive power for the short-term risk of heart failure in senior men, while TyG-WC showed superior predictive power for the short-term risk of heart failure in senior women.

A combined study based on a bidirectional cohort showed that women had a stronger correlation than men did between the TyG index and the risk of heart failure events [30], and another study found that men had a higher correlation than women did between TyG-derived indices and cardiovascular disease (CVD) [13]. Simultaneously, the impact of obesity levels on the population’s risk of heart failure varied according to gender.

Our research also revealed that there were gender differences in the relationships between several derived TyG indices and HF. These outcomes may be attributed to the higher prevalence of abdominal fat accumulation in women compared to men, making waist circumference a more accurate indicator of obesity in females. Numerous factors, such as physiological variations between men and women in terms of fat metabolism, hormone secretion, and body fat distribution, may have an impact on these gender variances. More investigation and learning are still needed to pinpoint the precise causes of these gender disparities.

### Effect of the longitudinal trajectory of derived TyG indexes on the occurrence of HF

The findings of the GBTM fitting demonstrate that irrespective of gender, the longitudinal trajectories of the TyG-BMI index stay comparatively steady over five years. The results of two cohort studies, with follow-up periods of five years [31] and ten years [32] among Chinese populations, likewise show that the longitudinal trajectories of the BMI and TyG index are stable at all levels. These findings imply that using baseline data accurately captures the effect of different TyG-BMI levels on the chance of developing HF over a period of five to 10 years. Regression analysis results indicated that within 5 years, those with a TyG-BMI maintained at 141.17–145.16 were more likely to experience heart failure than those with a TyG-BMI maintained at 173.84–176.72. This further supported this study’s findings, suggesting that the use of single-measured TyG-BMI data can predict 5–10 years of HF risk, and highlighted the importance of maintaining a moderate TyG-BMI level in lowering the risk of HF.

Furthermore, when the TyG-WHtR equal or less than 3.29 and continues to decline, men suffering from the risk of heart failure have 1.8 times higher than men with TyG-WHtR levels of 3.68 to 3.77. In comparison to the general population, this group is more likely to suffer from unfavorable health problems such as wasting, sarcopenia, and cachexia, as well as mobility issues [33]. Therefore, when utilizing TyG-WHtR as an indicator for monitoring the risk of heart failure in a population, it is crucial to simultaneously focus on individuals with a single measurement of TyG-WHtR equal to or greater than 3.85 and those exhibiting persistently low TyG-WHtR levels with a declining trend during longitudinal monitoring. Early intervention measures should be implemented to prevent developing risk of HF and other adverse health outcomes. It has not been determined whether distinct longitudinal trajectories of the TyG-BMI or TyG-WC index affect the risk of heart failure in the female population. It is noteworthy that there is now a paucity of research on the relationship between heart failure in the older population and longitudinal changes in the derived TyG index. Thus, additional research is required in the future to validate the results of this study.

### Strengths, limitations and future directions

The study’s strengths lie in the following: First, the data were obtained from the National Basic Public Health Examination project, which was convenient for the promotion and verification of the study findings in the community population; second, serial follow-up data resources are beneficial for future observation and assessment of the connection between derived TyG indexes and illness outcomes; third, this study examined for the first time any sex-specific variations in the relationship between derived TyG indexes and the risk of heart failure in the elderly Chinese, which offered novel viewpoints on how to create risk management plans tailored to the needs of various demographics. Nevertheless, the following are the limitations of this study: First of all, because this is an observational study, causality cannot be established, and there might be additional variables that could cause confusion. For instance, in our research,we did not account for the influence of socioeconomic status on the relationship between TyG combined obesity index and heart failure. Socioeconomic status is a recognized risk factor for cardiovascular disease occurrence, and this oversight may have led to an erroneous estimation of the association between the two in this study. Second, the study population, which was from a community in southwest China, had a unique age and geographic makeup, which limited how broadly the results could be applied. Thirdly, certain confounding factors, such as the medication status of the study population, were not adequately controlled for; additionally, while the research outcomes are based on diagnoses from healthcare professionals, there still exist potential errors like diagnostic inaccuracies, recording mistakes, which could introduce biases in the study, leading to erroneous estimations of the relationship between the derived TyG index and the risk of heart failure occurrence. Finally, the study’s index only takes into account five years changes. Increased sample size, more thorough epidemiological data collection, and analysis of long-term monitoring data can all help future studies better understand the relationship between novel glycemic and lipid metabolic markers and heart failure.

## Conclusion

This study analysed the association between the derived TyG indices incorporating obesity indicators in relation to the risk of HF in an elderly population in a community setting. After adjustment for additional risk factors, the study found a non-linear association between TyG-BMI and TyG-WC indices with the risk of heart failure in elderly population. Additionally, there was a significant positive correlation between the occurrence of HF and TyG-WHtR levels exceeding a certain threshold. Although this study has some limitations, it provides compelling evidence to support the use of the derived TyG index as a valid tool to identify the risk of developing HF in older adults over a five-year period.

## Supplementary Information

Additional file 1.

Additional file 2.

## Data Availability

The data that support the findings of this study are available from corresponding author but restrictions apply to the avail-ability of these data, which were used under license for the current study, and so are not publicly available. Data are however available from the authors upon reasonable request.

## Abbreviations

AIC: Akaike information criterion
ALT: Alanine Aminotransferase
AST: Aspartate Aminotransferase
AUC: Area under curve
AvePP: Average posterior probabilities
BMI: Body Mass Index
CI: Confidence intervals
CVD: Cardiovascular disease
DBP: Diastolic Blood Pressure
D/ND: Disease/Non-disease
GBTM: Group-based trajectory model
Hb: Hemoglobin
HDL-C: High-Density Lipoprotein Cholesterol
HF: Heart failure
HP: Hypertension
HRs: Hazard ratios
ICD: International Classification of Diseases
LDL-C: Low-Density Lipoprotein Cholesterol
OCC: Odds of correct classification
PLT: Platelet Count
RCS: Restricted cubic spline
RERI: Relative excess risk estimation
SBP: Systolic Blood Pressure
SCR: Serum creatinine
T2D: Type 2 Diabetes
TBIL: Total Bilirubin
TyG: Triglyceride-glucose
TyG-BMI: Triglyceride-glucose-body mass
TyG-WC: Triglyceride-glucose-waist circumference
TyG-WHtR: Triglyceride-glucose-waist-to-height
WC: Waist Circumference
WBC: White Blood Cell Count
WHtR: Waist-to-Height Ratio
10k PY: 10,000 person-years
