# Supplementary material for: The association between derived TyG index and the risk of heart failure in the elderly population: a prospective cohort study from 2017 to 2023
Source: BMC Public Health. 2025 Mar 4;25:863. doi: 10.1186/s12889-025-22046-2 (PMC11877815; doi:10.1186/s12889-025-22046-2)
Supplement: Supplementary file 1 — Additional file 1. [file 12889_2025_22046_MOESM1_ESM.docx]

**Detail of Hongguang Elderly Health Examination Cohort**

This study utilizes data from the Hongguang Elderly Health Examination Cohort. China's National Basic Public Health Service Project began providing annual health examinations to those 65 years of age and above in 2009. Since 2017, Chengdu Pidu District Hongguang Community Health Service Center has standardized the management of physical examination data collected by the National Basic Public Health Service Project, and an independent database has been formed.This health service center serves 14 community units, comprising 9 urban units and 5 rural units. The area has a registered permanent population of 26,516 adults aged 60 and above, with a male-to-female ratio of 1:1.16.

This ongoing, multi-community prospective cohort study primarily targets Chinese adults aged 60 and above. The objective was to investigate the health status of this population and to screen and verify the physiological and metabolic indicators for early risk prediction of common major chronic diseases in the elderly through continuous health examinations provided by basic public health services.

**Study Population:**

Participant recruitment was conducted in Hongguang Town, Pidu District, Chengdu City, Sichuan Province. Hongguang Town, a suburban area of Chengdu, is undergoing urbanization, and therefore, this region encompasses both urban and rural representative populations. Eligibility required participants:(1) to have completed at least once health check-up under the basic public health services during the study period; (2) have resided in the study area for at least six months.In addition, those with severe cardiovascular and cerebrovascular diseases at baseline and those who were unable to complete the physical examination for various reasons were excluded. The cohort finally comprises 12,577 adults aged around 60 and above from 14 communities, representing 47.43% of the local age-eligible population. The male-to-female ratio is 1:1.23.

**Data Collection**

The cohort was established between 2017 and 2023. Baseline data were collected through health check-ups, including demographic information(age, sex, ethnic, occupation, address, etc.), general health examinations(height, weight, waist, fasting blood glucose, triglyceride, total cholesterol, diastolic blood pressure, white blood cell, platelet, low-density lipoprotein cholesterol and high-density lipoprotein cholesterol,serum creatinine, etc.), lifestyle factors(exercise frequency, diet, smoking status, drinking status, occupational disease, ), health status(hospitalization, disease history, surgery history, etc.) and medication use, and health assessments(health self appraisal, self-assessment of cognitive function and emotion, etc.). During the study period, after signing a confidentiality agreement with Sichuan Provincial Health Information Center, Sichuan Provincial Health Information Center matched physical examination data with the disease diagnosis information system on the first page of medical records to collect the first confirmed information of chronic noncommunicable diseases of participants to confirm the occurrence and outcome of diseases of participants.

**Ethics approval and informed consent**

The research protocol was approved by the Medical Ethics Committee of West China Fourth Hospital of Sichuan University (Approval Number: Gwll2024175). As a secondary analysis of health facility data, all personal identifiers were removed, thus exempting it from the requirement for individual-level informed consent.
