# Supplementary material for: The association between derived TyG index and the risk of heart failure in the elderly population: a prospective cohort study from 2017 to 2023
Source: BMC Public Health. 2025 Mar 4;25:863. doi: 10.1186/s12889-025-22046-2 (PMC11877815; doi:10.1186/s12889-025-22046-2)
Supplement: Supplementary file 2 — Additional file 2. [file 12889_2025_22046_MOESM2_ESM.docx]

**Appendix：**

**First part: Assessment of additive and multiplicative interactions**

Multiplicative interaction and biological interaction were estimated by adding the cross-product interaction term and relative excess risk estimation (RERI) in the Cox regression model, respectively. Biological interactions in evaluation should be based on the sum of the scale^[[1]](#footnote-0),^^[[2]](#footnote-1)^ rather than multiplying the scale, so we used relative excess risk due to interaction (RERI) to estimate biological interactions. The RERI is the excess risk attributed to interaction relative to the risk without exposure. In the absence of additive interactions, RERI is equal to zero. In the present study, we refined the criteria as either a statistically significant *P*_for interaction_ <0.05 to indicate multiplicative interaction or 95% confidence intervals for the RERI that did not contain zeros to indicate biological interactions^[[3]](#footnote-2)^. Specifically, when RERI > 0, this signifies that the combined effects of obesity indexes and TyG on HF incidence exceed the sum of their individual effects, suggesting synergistic effects. Conversely, if RERI<0, it indicates that the combined effects are smaller than the sum of the individual effects of obesity indexes and TyG.

Table S1 Results of the interaction analysis

| Var1 | Var2 | *HR*(95%*CI*) | *P_for interaction_* | RERI(95%*CI*) | *P_for interaction_* |
| --- | --- | --- | --- | --- | --- |
| TyG_(_p_50)_ | |  |  |  |  |
|  | BMI(kg/m^2^) | 1.55(0.89,2.68) | 0.118 | 0.38(-0.10,0.86) | 0.120 |
|  | WC(cm) | 1.40(0.80,2.48) | 0.242 | 0.28(-0.25,0.81) | 0.297 |
|  | WHtR | 1.21(0.61,2.38) | 0.59 | 0.12(-0.52,0.76) | 0.712 |
| TyG_(_p_75)_ | |  |  |  |  |
|  | BMI(kg/m^2^) | 1.54(0.77,3.08) | 0.222 | 0.35(-0.24,0.94) | 0.245 |
|  | WC(cm) | 1.43(0.70,2.92) | 0.324 | 0.28(-0.35,0.91) | 0.389 |
|  | WHtR | 1.78(0.66,4.78) | 0.252 | 0.39(-0.28,1.07) | 0.252 |

**Abbreviation**: TyG: Triglyceride-glucose Index; BMI: Body Mass Index; WC: Waist Circumference Index; WHtR: Waist-to-Height Ratio Index; **Notes**: Each variable was converted into a binary variable according to the following principles to facilitate the exploration of interaction effects between variables:1) TyG index was converted to binary variables according to the 50th percentile and 75th percentile, respectively.;2) BMI was transformed into a binary variable according to whether it was 24 kg/m^2^ or greater;3) WC was transformed into a dichotomous variable according to whether it was 85 cm or greater for men and 80 cm or greater for women;4) WHtR was converted to a dichotomous variable according to whether it was greater than or equal to 0.5.

**Second part: Graphs and tables supplement**


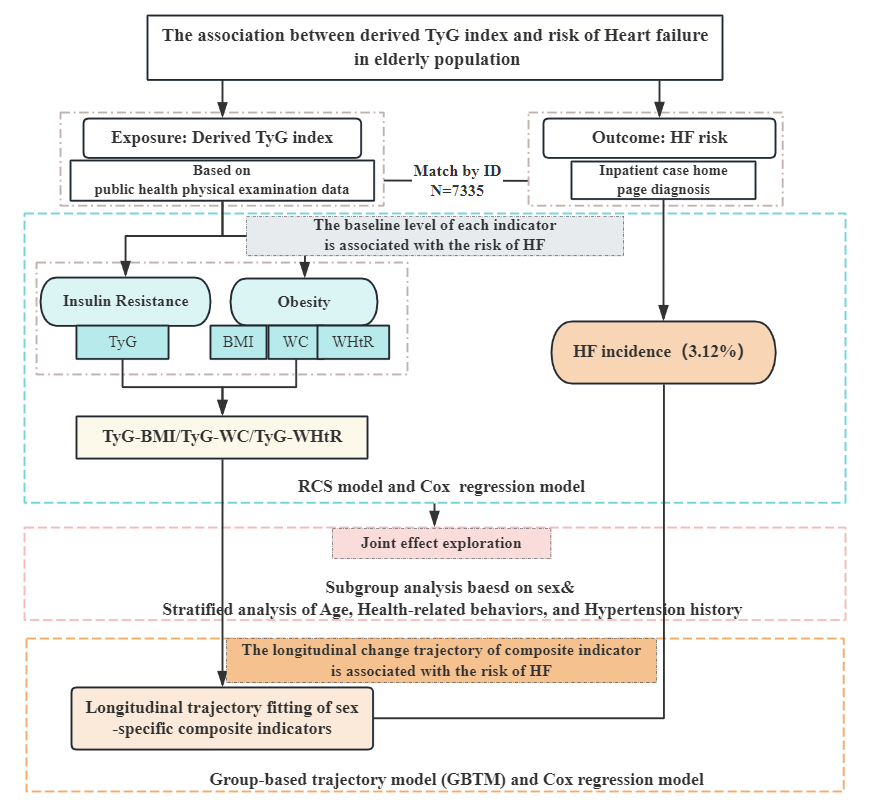


Figure S1. Research Technology Roadmap.


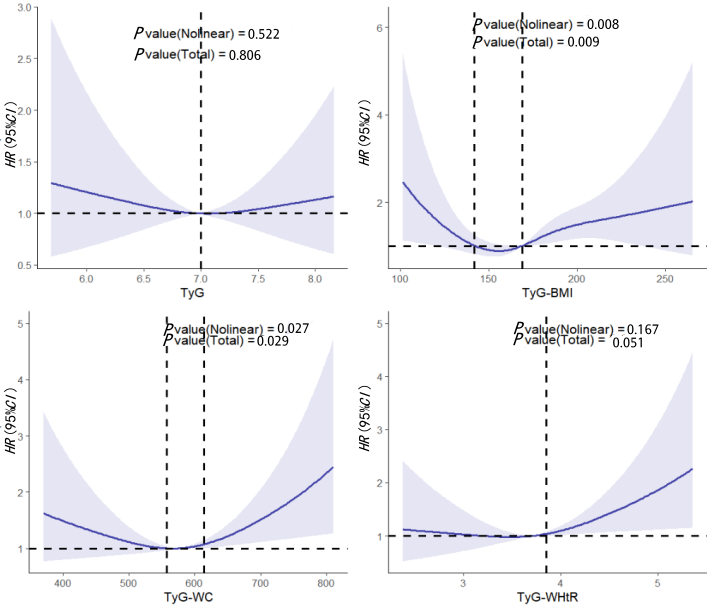


Note：The relationships between HF and TyG, TyG-WC and TyG-WHtR were well fitted in the three-node model based on the AIC criterion; however, the TyG-BMI index demonstrated a superior model fit with four nodes. The matching AIC values that reached the minimum value were 3542.4, 3536.97, 3535.92, and 3533.13.

Figure S2. Dose-response relationship between TyG and derived TyG indices and the risk of HF.

Table S2 RCS truncation value of TyG and its derived index

| Variable Names | Cut off value I | Cut off value II | G1 | G2 | G3 |
| --- | --- | --- | --- | --- | --- |
| TyG | 7 | - | <7 | >=7 | - |
| TyG-BMI | 142 | 169 | <142 | 142>=&<169 | >=169 |
| TyG-WC | 557 | 614 | <557 | 557>=&<614 | >=614 |
| TyG-WHtR | 3.85 | - | <3.85 | >=3.85 | - |

Abbreviations:TyG: Triglyceride-glucose index; TyG-BMI: Triglyceride-glucose body mass index; TyG-WC: Triglyceride-glucose waist circumference index; TyG-WHtR: Triglyceride-glucose waist-to-height ratio index.


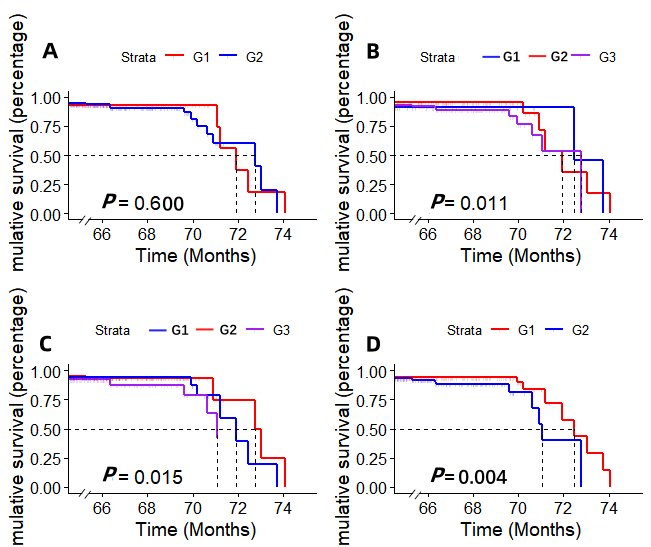


Note: A, B, C, D respectively for TyG, TyG - BMI, TyG - WC and TyG - WHtR K-M curve

Figure S3. Kaplan-Meier survival curves for baseline TyG and derived TyG indices.


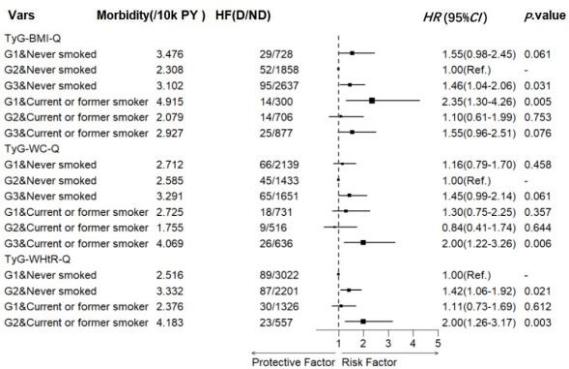

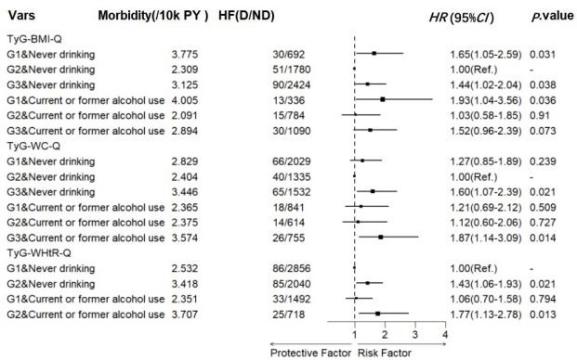


Abbreviations:G1:Group1(TyG-BMI:<142;TyG-WC:<557;TyG-WHtR:<3.85);G2:Group2(TyG-BMI:142>=&<169;TyG-WC:557>=&<614;TyG-WHtR:>=3.85); G3: Group 3(TyG-BMI:>=169;TyG-WC:>=614).

Figure S4. Forest plot of joint analysis based on smoking and different alcohol consumption habits

**Third part： Detail of GBTM**

3.1 Principles for selecting the optimal model

The longitudinal trajectories were fitted using a censored normal distribution. The number of groups was determined based on the best Bayesian information criterion, while the shape was determined by selecting the highest-order polynomial parameters with a p-value less than 0.05. Additionally, to prevent a non-parsimonious final model, the number of samples assigned to each trajectory group was constrained to be more than 5%. This approach ensured a coherent and informative model while avoiding excessive complexity^[[4]](#footnote-3)^. When fitting the longitudinal trajectory using GBTM, data from only two measurements were disqualified in order to guarantee a more consistent longitudinal trajectory. The final model's adequacy was evaluated through diagnostic procedures:(1) average posterior probabilities (AvePP) >=0.7;(2) odds of correct classification (OCC) >=5; (3) observed probability should have a proportion that is similar to the proportion of estimated probabilities from the model.To aid in a clear and intuitive interpretation of the outcomes, the names of the trajectory groups were then assigned by the visual pattern that the trajectory presented.

Based on the gender subgroup analysis results, separate trajectory models were fitted for TyG-WHtR in men and TyG-WC in women. Additionally, given that BMI is widely used to determine the degree of obesity in a population, we investigate the longitudinal trajectory of the TyG-BMI index in men and women. **Table S3** provides specifics on each GBTM model's parameters.

Table S3 Trajectory model parameters

| Vars | Class | Parameter | *β* | *S.E* | *P* | *Ek* |
| --- | --- | --- | --- | --- | --- | --- |
| Male | |  |  |  |  |  |
| TyG-BMI | |  |  |  |  | 0.88 |
|  | Low level&wave | Intercept | 145.76 | 0.970 | <0.001 |  |
|  |  | Linear | -8.97 | 1.650 | <0.001 |  |
|  |  | Quadratic | 4.24 | 0.790 | <0.001 |  |
|  |  | Cubic | -0.52 | 0.100 | <0.001 |  |
|  | Medium level&wave | Intercept | 176.04 | 0.900 | <0.001 |  |
|  |  | Linear | -3.06 | 1.440 | 0.34 |  |
|  |  | Quadratic | 1.96 | 0.680 | 0.004 |  |
|  |  | Cubic | -0.27 | 0.090 | 0.002 |  |
|  | High level&slight increase | Intercept | 211.66 | 1.200 | <0.001 |  |
|  |  | Linear | 1.05 | 0.310 | <0.001 |  |
| TyG-WHtR | |  |  |  |  | 0.84 |
|  | Low level&reduction | Intercept | 3.21 | 0.020 | <0.001 |  |
|  |  | Linear | -0.03 | 0.004 | <0.001 |  |
|  | Medium level&maintence | Intercept | 3.72 | 0.020 | <0.001 |  |
|  |  | Linear | 0.04 | 0.010 | 0.004 |  |
|  |  | Quadratic | -0.01 | 0.002 | 0.001 |  |
|  | High level&regression | Intercept | 4.29 | 0.030 | <0.001 |  |
|  |  | Linear | 0.16 | 0.020 | <0.001 |  |
|  |  | Quadratic | -0.03 | 0.004 | <0.001 |  |
| Female | |  |  |  |  |  |
| TyG-BMI | |  |  |  |  | 0.88 |
|  | Low level&wave | Intercept | 147.67 | 0.850 | <0.001 |  |
|  |  | Linear | -6.02 | 1.470 | <0.001 |  |
|  |  | Quadratic | 3.15 | 0.700 | <0.001 |  |
|  |  | Cubic | -0.40 | 0.090 | <0.001 |  |
|  | Medium level&maintenance | Intercept | 179.05 | 0.640 | <0.001 |  |
|  |  | Linear | 0.89 | 0.160 | <0.001 |  |
|  | High level&maintenance | Intercept | 219.05 | 1.020 | <0.001 |  |
|  |  | Linear | 0.84 | 0.260 | 0.001 |  |
| TyG-WC | |  |  |  |  | 0.84 |
|  | Low level&reduction | Intercept | 516.41 | 2.140 | <0.001 |  |
|  |  | Linear | -5.64 | 0.610 | <0.001 |  |
|  | Medium level&maintenance | Intercept | 603.18 | 2.530 | <0.001 |  |
|  |  | Linear | 3.03 | 1.880 | 0.106 |  |
|  |  | Quadratic | -0.92 | 0.340 | 0.007 |  |
|  | High level&regression | Intercept | 702.68 | 4.080 | <0.001 |  |
|  |  | Linear | 8.06 | 3.170 | 0.011 |  |
|  |  | Quadratic | -1.30 | 0.580 | 0.253 |  |

**Abbreviation**: TyG-BMI: Triglyceride-glucose Body Mass Index; TyG-WC: Triglyceride-glucose Waist Circumference Index; TyG-WHtR: Triglyceride-glucose Waist-to-Height Ratio Index; Ek: Relative entropy value，(Ek>0.7, model’s classification accuracy is acceptable.)

3.2 The trajectory fluctuation range of each group

Over six years, the TyG-BMI index remained stable for both genders. In the female population, the trajectory of the TyG-WC index exhibited a similar pattern to that of the TyG-WHtR index in males, showing three distinct groups: a low-level decline group, a medium-level maintenance group, and a high-level regression group.The fluctuation range and change over time of each indicator track group for the male and female elderly population are shown in **Table S4**.**Figure S5** shows the results of the trajectory fitting for males, while the results for females are presented in **Figure S6**.

Table S4. Precise description of the trajectory in each group

| Group Classification | *‾x_base_*,*‾(x_min_*,*‾x_max_*) | *AvePP*（%） | *OCC* | *π*_j_（%） | *P_j_*（%） |
| --- | --- | --- | --- | --- | --- |
| **Male** |  |  |  |  |  |
| *TyG-BMI* |  |  |  |  |  |
| Low level&wave | 145.16(141.17,145.16) | 0.94 | 30.33 | 0.36 | 0.36 |
| Medium level&wave | 174.87(173.84,176.72) | 0.94 | 15.99 | 0.49 | 0.49 |
| High level&slight increase | 196.05(196.05,206.59) | 0.95 | 110.28 | 0.15 | 0.16 |
| *TyG-WHtR* |  |  |  |  |  |
| Low level&reduction | 3.29(3.04,3.29) | 0.93 | 25.73 | 0.35 | 0.35 |
| Medium level&maintence | 3.71(3.68,3.77) | 0.93 | 13.39 | 0.48 | 0.48 |
| High level&regression | 4.02(4.02,4.26) | 0.91 | 53.77 | 0.17 | 0.17 |
| **Female** |  |  |  |  |  |
| *TyG-BMI* |  |  |  |  |  |
| Low level&wave | 147.71(144.24,147.87) | 0.95 | 34.76 | 0.35 | 0.35 |
| Medium level&maintenance | 176.43(176.43,182.44) | 0.94 | 17.39 | 0.48 | 0.48 |
| High level&maintenance | 207.58(207.58,215.72) | 0.94 | 79.70 | 0.17 | 0.17 |
| *TyG-WC* |  |  |  |  |  |
| Low level&reduction | 527.08(489.17,527.08) | 0.93 | 25.71 | 0.35 | 0.35 |
| Medium level&maintenance | 597.89(587.76,601.34) | 0.92 | 13.26 | 0.48 | 0.48 |
| High level&regression | 660.65(660.65,682.73) | 0.93 | 61.60 | 0.17 | 0.17 |

**Abbreviation**: *AvepP*: the average posterior probability of assignment; *OCC*: odds of correct classification.


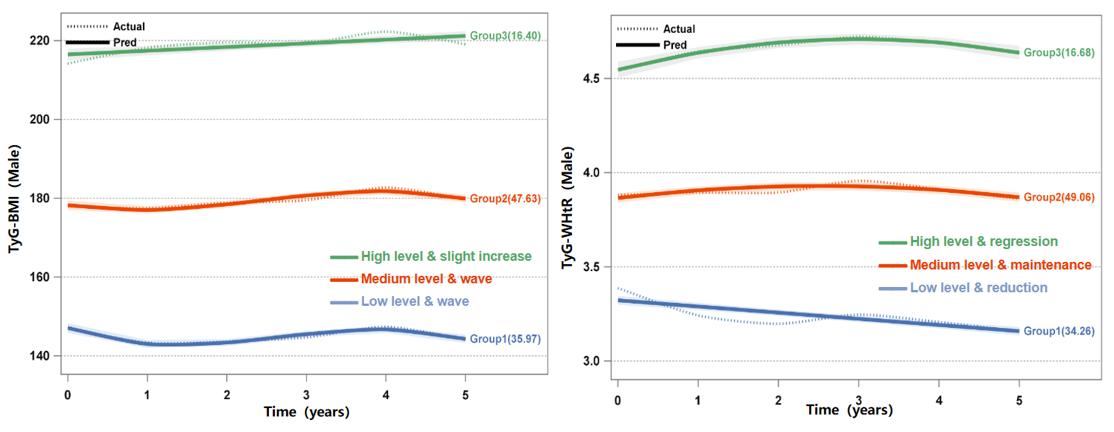


Figure S5. TyG-BMI and TyG-WHtR longitudinal trajectory of the male population


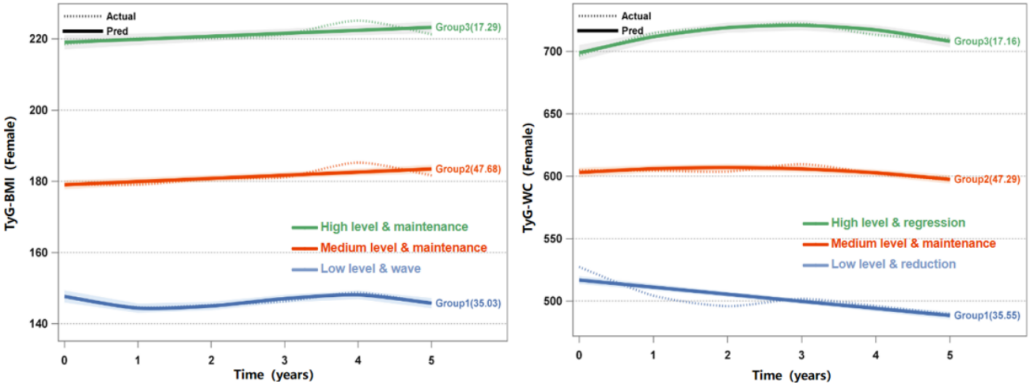


Figure S6. TyG-BMI and TyG-WC longitudinal trajectory of female population

3.3 The trajectory fluctuation range of each group

The results of Cox regression model showed that the TyG-BMI index low-level wave group in males had a 137% higher risk of HF than the medium-level wave group. Additionally, compared to the medium-level maintenance group, the low-level reduction group of TyG-WHtR index had a 1.8-fold increased risk of HF. Nonetheless, across the different longitudinal trajectories of TyG-BMI and TyG-WC in women, there were no appreciable variations in the risk of HF.Details are shown in **Table S5**.

Table S5. Survival analysis of different trajectory groups of derived TyG indices in relation to HF

| Group Classification | N | HF Incidence（%） | *HR*(95%*CI*)^a^ |
| --- | --- | --- | --- |
| **Male** |  |  |  |
| *TyG-BMI* |  |  |  |
| Low level&wave | 633 | 5.53 | 2.37（1.39，4.07） |
| Medium level&wave | 866 | 2.54 | Ref. |
| High level&slight increase | 269 | 5.20 | 1.88（0.95，3.74） |
| *TyG-WHtR* |  |  |  |
| Low level&reduction | 620 | 5.00 | 1.80（1.07，3.05） |
| Medium level&maintence | 850 | 3.06 | Ref. |
| High level&regression | 298 | 4.70 | 1.39（0.71，2.71） |
| **Female** |  |  |  |
| *TyG-BMI* |  |  |  |
| Low level&wave | 837 | 3.94 | 0.71（0.45，1.12） |
| Medium level&maintenance | 1145 | 4.45 | Ref. |
| High level&maintenance | 415 | 4.10 | 0.99（0.57，1.72） |
| *TyG-WC* |  |  |  |
| Low level&reduction | 835 | 3.71 | 0.69（0.44，1.09） |
| Medium level&maintenance | 1148 | 4.62 | Ref. |
| High level&regression | 414 | 4.11 | 0.94（0.54，1.63） |

Note:The Cox model was adjusted for age, gender, history of hypertension, smoking history, alcohol consumption, Scr, SBP, DBP, HDL-C, and LDL-C.

**Fourth part：Specific measurement methods for the main research variables**

4.1 Height, Weight, Body Mass Index and Waist circumference

Height: The examinee should remove shoes, hat, and outerwear. Stand upright with eyes facing forward, chest out, abdomen in, arms naturally hanging down, fingers together, heels together and toes apart approximately 60 degrees, back against the pillar of the stadiometer, ensuring that the heels, buttocks, and shoulders of both feet touch the pillar. The measurer moves the stadiometer until the top of the head touches it, reads and records the measurement in centimeters (cm).

Weight: Before the examination, the weight scale should be zeroed. The examinee should remove shoes, hat, and outerwear. They should empty their bladder in the morning on an empty stomach, stand naturally and steadily in the center of the weight scale platform, hands naturally hanging down, avoiding shaking or pressure, accurately read and record the measurement in kilograms (kg).

Body Mass Index (BMI) = Weight (kg) / Height squared (m2).

Waist circumference(WC): The horizontal position at the midpoint between the lower edge of the rib arch on the mid-axillary line and the iliac crest line is the measurement point. Mark both sides, repeat the measurement twice, record the average value with one decimal place, and the unit is in centimeters (cm).

4.2 Measurement of Blood Glucose and Lipid Indices

Fasting blood glucose(FPG) and lipid indices(total cholesterol , triglycerides , and high-density lipoprotein cholesterol,etc. ) were obtained through clinical blood tests, the specific preparation and blood sample collection and processing methods for the subjects are as follows:

(1) Subjects should be in a stable metabolic state for at least 2 weeks, maintaining their normal dietary habits and stable weight.

(2) Subjects should avoid vigorous physical activity within 24 hours before sample collection.

(3) Subjects should fast for about 12 hours before sample collection.

(4) Venous blood samples are taken for testing. Before blood draw, subjects should sit quietly for at least 5 minutes. Unless under special circumstances, subjects should be seated during blood collection.

(5) The tourniquet should not be applied for more than 1 minute during venipuncture.

(6) Blood samples should be sealed and agitation should be avoided as much as possible.

(7) Serum should be used for analysis samples, and blood samples should be centrifuged within 1-2 hours.

(8) Analyze serum samples promptly to avoid sample storage. If storage is necessary, samples should be kept sealed. Short-term storage (within 3 days) can be done at 4°C, while long-term storage should be below -70°C.

(9) Enzymatic methods are commonly used in clinical laboratories to measure FPG and lipid indices.

**Fifth part：HR of the remaining variables in the Adjusted Model**

Table S6. HR of the remaining variables in the Adjusted Model

| Variable Name | *HR*(95%*CI*) | Variable Name | *HR*(95%*CI*) |
| --- | --- | --- | --- |
| TyG-Q | | | |
| Age | 1.08(1.05-1.10) | Scr | 1.53(0.64-3.66) |
| gender | 1.15(0.79-1.66) | SBP | 1.01(1.00-1.02) |
| History of Hypertension | 2.25(1.55-3.28) | DBP | 0.99(0.98-1.01) |
| Smoking History | 1.18(0.78-1.79) | HDL-C | 0.88(0.56-1.37) |
| Alcohol Consumption | 1.00(0.69-1.46) | LDL-C | 1.09(0.90-1.31) |
| TyG-BMI-Q | | | |
| Age | 1.08(1.05-1.10) | Scr | 1.65(0.69-3.94) |
| gender | 1.15(0.79-1.66) | SBP | 1.01(1.00-1.02) |
| History of Hypertension | 2.29(1.57-3.33) | DBP | 0.99(0.98-1.01) |
| Smoking History | 1.17(0.77-1.77) | HDL-C | 0.90(0.57-1.43) |
| Alcohol Consumption | 0.99(0.67-1.44) | LDL-C | 1.08(0.90-1.29) |
| TyG-WC-Q | | | |
| Age | 1.08(1.05-1.10) | Scr | 1.58(0.66-3.78) |
| gender | 1.14(0.79-1.65) | SBP | 1.01(1.00-1.02) |
| History of Hypertension | 2.20(1.51-3.20) | DBP | 0.99(0.98-1.01) |
| Smoking History | 1.17(0.77-1.77) | HDL-C | 0.94(0.60-1.48) |
| Alcohol Consumption | 1.01(0.69-1.48) | LDL-C | 1.06(0.89-1.28) |
| TyG-WHtR-Q | | | |
| Age | 1.07(1.05-1.10) | Scr | 1.61(0.67-3.85) |
| gender | 1.07(0.74-1.55) | SBP | 1.01(1.00-1.02) |
| History of Hypertension | 2.21(1.52-3.22) | DBP | 0.99(0.98-1.01) |
| Smoking History | 1.17(0.77-1.77) | HDL-C | 0.99(0.63-1.54) |
| Alcohol Consumption | 1.02(0.70-1.48) | LDL-C | 1.05(0.88-1.26) |

Abbreviations: *CI*, confidence interval; *HR*, hazard ratio；Scr: Serum Creatinine; SBP: Systolic Blood Pressure; DBP: Diastolic Blood Pressure; HDL-C: High-Density Lipoprotein Cholesterol; LDL-C: Low-Density Lipoprotein Cholesterol.

1. Rothman K J, Greenland S, Lash T L. Modern epidemiology[M]. Philadelphia: Wolters Kluwer Health/Lippincott Williams & Wilkins, 2008. [↑](#footnote-ref-0)
2. Hosmer D W, Lemeshow S. Confidence interval estimation of interaction[J]. Epidemiology, 1992: 452-456. [↑](#footnote-ref-1)
3. Knol M J, VanderWeele T J, Groenwold R H H, et al. Estimating measures of interaction on an additive scale for preventive exposures[J]. European journal of epidemiology, 2011, 26: 433-438. [↑](#footnote-ref-2)
4. Nagin D (1999) Analyzing developmental trajectories: a semi parametric, group-based approach. Psychol Meth 4, 139–157. [↑](#footnote-ref-3)
